# Investigation on Potential Correlation Between Small Nuclear Ribonucleoprotein Polypeptide A and Lung Cancer

**DOI:** 10.3389/fgene.2020.610704

**Published:** 2021-01-21

**Authors:** Maoxi Yuan, Chunmei Yu, Xin Chen, Yubing Wu

**Affiliations:** ^1^Department of Thoracic Surgery, Linyi Central Hospital, Linyi, China; ^2^Department of Thoracic Surgery, The People's Hospital of Feixian County, Linyi, China

**Keywords:** *SNRPA*, lung cancer, expression, prognosis, phosphorylation

## Abstract

*SNRPA* (small nuclear ribonucleoprotein polypeptide A) gene is essential for the pre-mRNA splicing process. Using the available datasets of TCGA or GEO, we aimed at exploring the potential association between the *SNRPA* gene and lung cancer by several online tools (such as GEIPA2, MEXPRESS, Oncomine) and bioinformatics analysis software (R or GSEA). *SNRPA* was highly expressed in the tissues of lung adenocarcinoma (LUAD) and lung squamous cell carcinoma tissue (LUSC), compared with control tissues. The high SNRPA expression was associated with a poor survival prognosis of LUAD cases, while the genetic alteration within *SNRPA* was linked to the overall survival prognosis of LUSC cases. There was a potential correlation between promoter methylation and the expression of *SNRPA* for LUAD. Compared with normal tissues, we observed a higher phosphorylation level at the S115 site of SNRPA protein (NP_004587.1) (*p* = 0.002) in the primary LUAD tissues. The potential ATR kinase of the S115 site was predicted. Besides, *SNRPA* expression in lung cancer was negatively correlated with the infiltration level of M2 macrophage but positively correlated with that of Follicular B helper T cells, in both LUAD and LUSC. The enrichment analysis of *SNRPA*-correlated genes showed that cell cycle and ubiquitin mechanism-related issues were mainly observed for LUAD; however, RNA splicing-related cellular issues were mainly for LUSC. In summary, the *SNRPA* gene was identified as a potential prognosis biomarker of lung cancer, especially lung adenocarcinoma, which sheds new light on the association between the spliceosomal complex component and tumorigenesis.

## Introduction

Lung cancer, a type of tumor that originates in the bronchial mucosa or glands of the lung tissue, shows a group of clinical symptoms, such as cough, blood in the sputum, wheezing, and chest pain, and so on (Duruisseaux and Esteller, 2018; Nasim et al., 2019). According to the different characteristics of histopathology, lung cancer can be divided into two main types, including non-small cell lung cancer (NSCLC) and small cell lung cancer (SCLC) (Duffy and O'Byrne, 2018; Testa et al., 2018; Friedlaender et al., 2019). There are two main distinct subtypes of NSCLC, namely lung adenocarcinoma (LUAD) and lung squamous cell carcinoma (LUSC) (Duffy and O'Byrne, 2018; Testa et al., 2018; Friedlaender et al., 2019). Considering the complicated pathogenesis of lung cancer, it is meaningful to explore the molecular mechanisms of lung cancer-associated oncogenes (Diaz-Lagares et al., 2016). In the present study, we aimed at investigating the potential mechanism of the *SNRPA* gene in the prognosis of LUAD and LUSC.

Small nuclear ribonucleoprotein polypeptide A (SNRPA) protein, encoded by *the SNRPA* gene located on chromosome 19q13.2, is implicated in the assembly of U1 small nuclear ribonucleoprotein (U1 snRNP) complex, and pre-mRNA splicing process (Bai et al., 2013; Singh and Singh, 2019; Subramania et al., 2019). However, there is still no publication analyzing the potential effect of the *SNRPA* in the prognosis of lung cancer. Some available online datasets can help in the identification of some clinical prognosis-related oncogenes. For instance, as a public funded project, the cancer genome atlas (TCGA) contains the expression, mutation, methylation, clinical datasets of more than 30 types of cancer (Chang et al., 2015). Also, gene expression omnibus (GEO) database including the expression data of different cancer patients (Clough and Barrett, 2016).

In this study, we utilized the online approaches and bioinformatics analysis software to explore the potential relationship between SNRPA and lung adenocarcinoma or squamous cell carcinoma. The possible molecular mechanisms of SNRPA in lung carcinogenesis were investigated from different aspects, including the gene expression difference, survival value, genetic mutation, DNA methylation, phosphorylation, immune cell infiltration, and enrichment analysis of *SNRPA*-related genes.

## Materials and Methods

### Expression Analysis

We analyzed the expression difference of *SNRPA* between lung cancer tissues and normal control tissues in the TCGA-LUAD (lung adenocarcinoma) or TCGA-LUSC (lung squamous cell carcinoma) cohorts by the “Expression DIY” module of an online tool of “gene expression profiling interactive analysis version 2” (GEIPA2) (http://gepia2.cancer-pku.cn/#analysis) (Tang et al., 2019). The data was visualized by a box plot. In addition, a violin plot showing the expression status of *SNRPA* among different pathological stages (stage I, II, III, and IV) was obtained as well. We used the “TCGAbiolinks” R package to download the “Fragments Per Kilobase of exon model per Million mapped fragments- upper quantile” (FPKM-UQ) standardized expression matrices of TCGA-LUAD and TCGA-LUSC cohorts, respectively. After the sorting and the logarithm base 2 (log2) transformation of expression matrix, we obtained the paired *SNRPA* expression data of the lung cancer and the corresponding para-carcinoma tissues (*n* = 57 for LUAD pair, *n* = 49 for LUSC pair). The “compare_means()” R function was then used for a Wilcoxon test, and the result was visualized by the “ggdotchart ()” of “ggpubr” R package [paired = T]. Besides, we utilized the MEXPRESS approach (https://mexpress.be/) (Koch et al., 2015, 2019) to investigate the correlation between *SNRPA* expression and a group of clinical factors (e.g., age, gender, ethnicity, race, residual tumor, histological type, eastern cancer oncology group, etc.) for the LUAD and LUSC cases in TCGA database, respectively. Benjamini-Hochberg-adjusted *p*-value of positive results in a Pearson test was provided.

Apart from the TCGA database, we also tried to pool the available online datasets through the Oncomine database (https://www.oncomine.org/resource/login.html) for a comprehensive evaluation of *SNRPA* expression difference between normal and lung adenocarcinoma or squamous cell lung carcinoma tissues. The *p*-value of the median-ranked analysis across different datasets was provided.

### Survival Curve Analysis

We analyzed the potential correlation between the *SNRPA* expression and the clinical prognosis of LUAD/LUSC cases in the TCGA database. We first utilized the “Survival Analysis” module of GEPIA2 (http://gepia2.cancer-pku.cn/#survival) to obtain the survival plot of overall survival (OS) with the *p*-value of the Log-rank test. The “Group Cutoff with median” was set for splitting the high/low- expression of SNRPA cohorts. Furthermore, we used the “survival” R package to perform the univariate and multivariate COX regression analyses for the overall survival assessment of LUAD and LUSC cases in TCGA. The factors, including the SNRPA expression, pathological stages (stage I, II, III/IV), gender (male, female), age (“>50,” “<=50”), race (white, non-white), were included. The data of *p*-value, HR (Hazard ratio), 95% CI (confidence interval) was yielded by the R functions of coxph () and summary (), while a forest plot was obtained through the “plot ()” R function.

Apart from the above TCGA-LUAD/LUSC cohorts, we also tried to collect the available lung cancer datasets (e.g., CAARRAY, GSE14814, GSE19188, GSE29013, GSE30219, GSE31210, GSE3141, etc.) for the overall survival (OS), first-progression (FP), post-progression survival (PPS) analyses using an online tool of Kaplan-Meier plotter (http://kmplot.com/analysis/index.php?p=service&cancer=lung) (Gyorffy et al., 2013). The clinical factors, including the gender, smoking history, stage, grade, AJCC (American Joint Committee on Cancer) stage t/n/m, surgery, radiotherapy, chemotherapy, were also considered in the subgroup analysis. Several recent literatures (Cui et al., 2020; Li et al., 2020a; Zhao et al., 2020) were referred. The array quality control with “exclude biased arrays (*n* = 2434)” and the “Auto select best cutoff” were set.

### Genetic Alteration Analysis

We explored the genetic alteration feature of *SNRPA* in the TCGA-LUAD/LUSC cohorts, using the cBioPortal web service (https://www.cbioportal.org/). The data of alteration frequency and alteration type were visualized by an “OncoPrint” module of cBioPortal. The OS and disease/progression-free survival (D/PFS) analyses of *SNRPA* genetic alteration were performed using lung cancer cases within TCGA-LUAD/LUSC cohorts.

### DNA Methylation Analysis

We utilized the MEXPRESS (Koch et al., 2015, 2019) approach to analyze the DNA methylation status of *SNRPA* for the cases of TCGA-LUAD/LUSC cohorts. The correlation between DNA methylation and gene expression of *SNRPA* was measured by a Pearson's test. Benjamini-Hochberg-adjusted *p* and corresponding r (correlation coefficients) values were shown. Also, the differences in the expression or promoter methylation of *SNRPA* between the normal and primary lung tumor tissues were analyzed using a UALCAN web-portal (http://ualcan.path.uab.edu/) (Chandrashekar et al., 2017).

### *SNRPA* Phosphorylation Analysis

Based on the lung adenocarcinoma dataset of clinical proteomic tumor analysis consortium (CPTAC), we used the UALCAN portal (http://ualcan.path.uab.edu/analysis-prot.html) (Chen et al., 2019; Cui et al., 2020) to analyze the difference of expression or phosphorylation level (S115 and T131 sites) of SNRPA protein (NP_004587.1) between normal tissue and primary lung adenocarcinoma cancer tissue. Using an open-access PhosphoNET web (http://www.phosphonet.ca/), the potential kinases can be predicted by the calculation of “Kinase Predictor V2” score. Thus, we utilized the PhosphoNET to analyze the phosphorylation status and the potential kinases of the two sites (S115 and T131) within the SNRPA protein. The information of predicted kinases with the highest score of “Kinase Predictor V2” was provided.

### Immune Cell Infiltration Analysis

We first applied the “Correlation Analysis” module of GEPIA2 (http://gepia2.cancer-pku.cn/#correlation) (Pan et al., 2019; Tang et al., 2019) to perform a pair-wise gene correlation analysis between *SNRPA* expression and the signatures of the following immune cells: monocytes, M1/2 macrophage, tumor-associated macrophages (TAMs), natural killer cell (NK cell), neutrophils, basophils, eosinophils, mast cell, dendritic cell, B cell, CD8^+^ T cell, follicular B helper T cell (Tfh), effector T-cell, Exhausted T-cell. The r and *p*-values in a Spearman's test were obtained. Referring to several relevant literatures (Cui et al., 2020; Li et al., 2020b; Zhang et al., 2020), we utilized the “Immune-gene” module of TIMER2.0 (http://timer.cistrome.org/) to further analyze the correlation between *SNRPA* expression and the immune infiltration levels of M2 Macrophage and Tfh for TCGA, under the algorithms of CIBERSORT, CIBERSORT-ABS, QUANTISEQ, or XCELL. The purity-adjusted Rho and *p*-values were yielded in a Spearman's test.

### *SNRPA*-Correlated Gene Analysis

We performed the cluster analysis of the *SNRPA-*correlated significant genes through the “TCGA analysis” module of UALCAN approach (http://ualcan.path.uab.edu/analysis.html). The heat maps containing the *SNRPA* positively or negatively correlated significant genes (top 4) in LUAD and LUSC were shown. We then analyzed the expression correlation between *SNRPA* and the selected genes in LUAD and LUSC. The race information of cases was indicated as well. Using a Venn tool (http://bioinformatics.psb.ugent.be/webtools/Venn/), we analyzed the difference of *SNRPA*-correlated genes between LUAD and LUSC, and obtained three gene lists, including the “LUAD/LUSC common,” “LUAD only,” and “LUSC only” genes. Further, we performed a “Kyoto Encyclopedia of Genes and Genomes” (KEGG) pathway analysis of the three gene lists using a “Database for Annotation, Visualization, and Integrated Discovery” (DAVID) online tool (https://david.ncifcrf.gov/) and a “ggplot2” R package. We also performed a “Gene ontology” (GO) enrichment analysis by a “clusterProfiler” R package and visualized the results of biological process, cellular component, and molecular function by the Microsoft EXCEL 2019 software. The cnetplots of MF in the groups of LUAD/LUSC, LUAD, LUSC, were also generated by the “netplots ()” R function. In addition, based on the median value of *SNRPA* expression, the expression matrix of TCGA-LUAD/LUSC cohorts was divided into “High-expression” and “Low-expression” groups. Then, we perform a “Gene Set Enrichment Analysis” (GSEA) with the setting of “High-expression vs. Low-expression.” The values of NES (normalized enrichment score), nominal *p* and FDR (false discovery rate) were obtained by the GSEA software (version 4.0.3), as in previous reports (Chen et al., 2020; Guo et al., 2020; Zhang et al., 2020). The R language software (version 3.6.1) was applied to run the above R packages or R functions.

## Result

### Expression Feature of *SNRPA*

**Figure 1:**
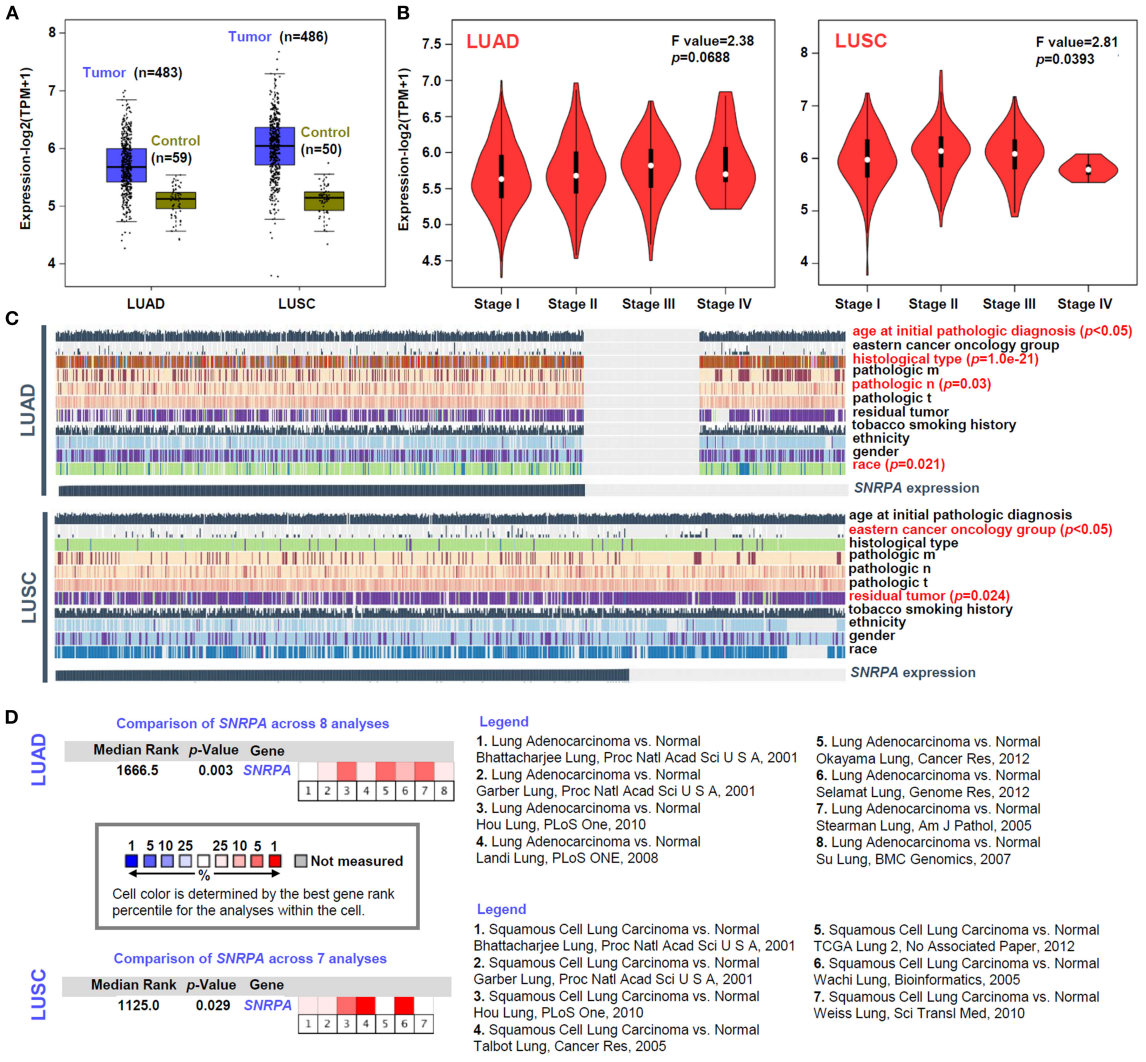
Expression analysis of *SNRPA*. **(A)** The *SNRPA* expression levels in the tumor and control tissues of TCGA-LUAD and TCGA-LUSC cohorts were analyzed by a GEPIA2 tool. **(B)** The expression levels of *SNRPA* among different pathological stages were analyzed as well. **(C)** The association between *SNRPA* expression and a series of clinical factors (e.g., age, gender, ethnicity, race, residual tumor, histological type, eastern cancer oncology group, etc.) was analyzed by MEXPRESS. **(D)** The expression difference of *SNRPA* between normal and LUAD/LUSC tissues was analyzed by pooling the collected datasets from Oncomine. The detailed dataset information and *p*-value were provided.

Based on the datasets of TCGA-LUAD and TCGA-LUSC cohorts, we analyzed the expression level of *SNRPA* between lung cancer and the para-carcinoma tissues. We first enrolled all lung cancer cases (*n* = 483 for LUAD; *n* = 486 for LUSC) as the “Tumor” group and observed a higher expression level of *SNRPA* in the “Tumor” group, compared with the “Control” group (Figure 1A). Then, we only extracted the dataset of lung cancer tissues and the corresponding para-carcinoma tissues and obtained similar results (Supplementary Figure 1, *n* = 57, *p* = 6.9e−11 for LUAD pair; *n* = 49, *p* = 3.2e−20 for LUSC pair). Furthermore, there was a positive correlation between the *SNRPA* expression and pathological stages (stage I~IV) of LUSC cases (Figure 1B, *p* = 0.0393), but not the LUAD cases (*p* = 0.0688). Despite this, we found that *SNRPA* expression in the LUCA cases was related to the factors of pathologic n (Figure 1C, *p* = 0.03), age at initial pathologic diagnosis (*p* < 0.05), histological type (*p* = 1.0e−21), race (*p* = 0.21). *SNRPA* expression in the LUSC cases was linked to the factors of the eastern cancer oncology group (Figure 1C, *p* < 0.05), residual tumor (*p* = 0.024) as well.

Besides, we tried to include the available datasets containing the lung adenocarcinoma and normal tissues from the Oncomine database for further investigation. As shown in Figure 1D, we include eight datasets for the pooling analysis of LUAD and seven datasets for that of LUSC. Also, the positive results were detected in both LUAD (Figure 1D, *p* = 0.003) and LUSC (*p* = 0.029). Hence, the above confirmed the high expression of *SNRPA* in lung cancer tissues, which indicates the potential role of *SNRPA* in the etiology of LUAD or LUSC.

### Prognostic Value of *SNRPA*

**Figure 2:**
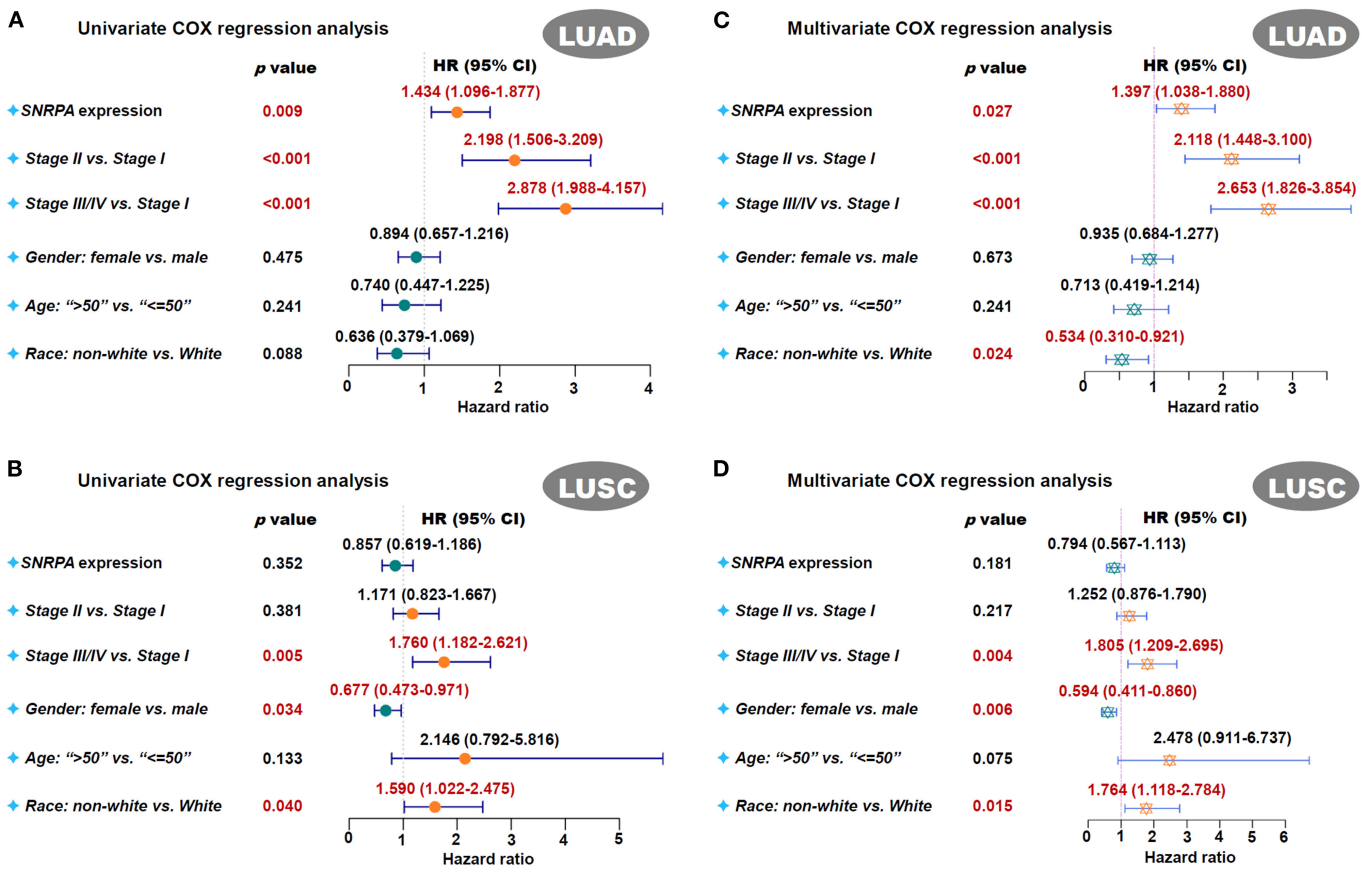
Univariate and multivariate COX regression analysis of *SNRPA*. We performed the univariate and multivariate COX regression analyses for the overall survival assessment of LUAD **(A,B)** and LUSC **(C,D)** cases in TCGA. The factors, including the *SNRPA* expression, pathological stages, gender, age, race, were included. The forest plots with *p*-value, HR, 95% CI were provided.

Next, we analyzed the potential relationship between *SNRPA* expression pattern and the clinical prognosis of lung cancer cases. As shown in survival plots of Supplementary Figure 2, compared with the *SNRPA* low expression group, there was a lower rate of overall survival (OS) in the high expression group of TCGA-LUAD (Log-rank test, *p* = 0.00028), but not TCGA-LUSC (*p* = 0.95). Also, we observed a similar correlation of *SNRPA* expression and clinical prognosis in both univariate (Figure 2A, HR = 1.434, *p* = 0.009 for LUAD; Figure 2B, *p* = 0.352 for LUSC) and multivariate (Figure 2C, HR = 1.397, *p* = 0.027 for LUAD; Figure 2D, *p* = 0.181 for LUSC) COX regression analyses for TCGA-LUAD/LUSC cohort.

Besides the above TCGA datasets, we also pooled a series of CAARRAY or GEO datasets for the preformation of OS, FP, and PPS analyses. As shown in Supplementary Table 1, there was a positive correlation between *SNRPA* high expression and the worse survival prognosis of lung cancer cases in the subgroups of “female,” “exclude those never smoked,” “only those never smoked,” “pathologic stage I,” and “only surgical margins negative” (all HR > 1, *p* < 0.05). In terms of lung cancer type, the high expression of *SNRPA* was related to the low rates of OS (Supplementary Figure 3A, *p* = 2.0e−13), FP (Supplementary Figure 3B, *p* = 3.1e−05), and PPS (Supplementary Figure 3C, *p* = 0.011) for LUAD cases. In addition, the highly expressed *SNRPA* was associated with a worse prognosis of OS (Supplementary Figure 3A, *p* = 0.015), but a better prognosis of FP (Supplementary Figure 3B, *p* = 0.012) for LUSC cases. Thus, SNRPA may be a novel prognostic biomarker of lung adenocarcinoma.

### Genetic Alteration of *SNRPA*

**Figure 3:**
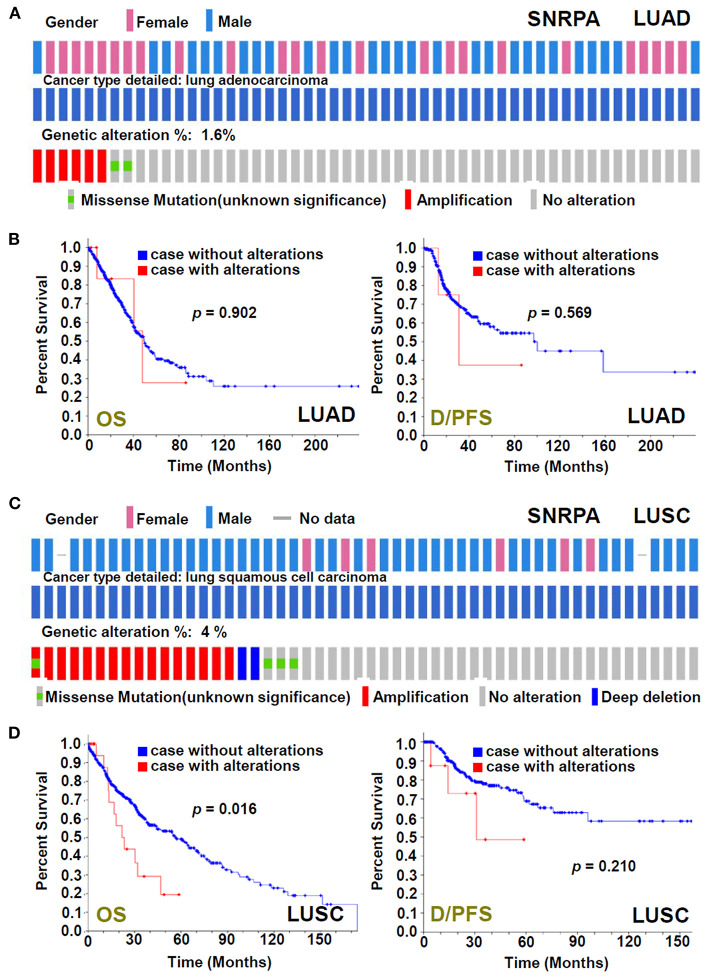
Genetic alteration analysis of *SNRPA*. We analyzed the genetic alteration feature of *SNRPA* and performed the OS and disease/progression-free survival (D/PFS) analyses of cases within TCGA-LUAD **(A,B)** and TCGA-LUSC **(C,D)** cohorts, respectively. The alteration frequency, alteration type, and survival curve were shown.

The genetic alteration status of the *SNRPA* gene in the TCGA-LUAD/LUSC cohorts was analyzed. As shown in Figure 3A, we only observed a genetic alteration rate of 1.6% with the type of “missense mutation” and “amplification” for the LUAD cases. There was no correlation between the genetic alteration of *SNRPA* and the clinical outcomes of lung adenocarcinoma cases (Figure 3B). However, for LUSC cases, we observed the 4% alteration rate with the type of “missense mutation,” “amplification,” and “deep deletion” (Figure 3C), and the correlation between the genetic alteration of *SNRPA* and the worse OS prognosis (Figure 3D, *p* = 0.016), suggesting the potential involvement of *SNRPA* genetic alteration in the clinical prognosis of lung squamous cell carcinoma.

### DNA Methylation of *SNRPA*

**Figure 4:**
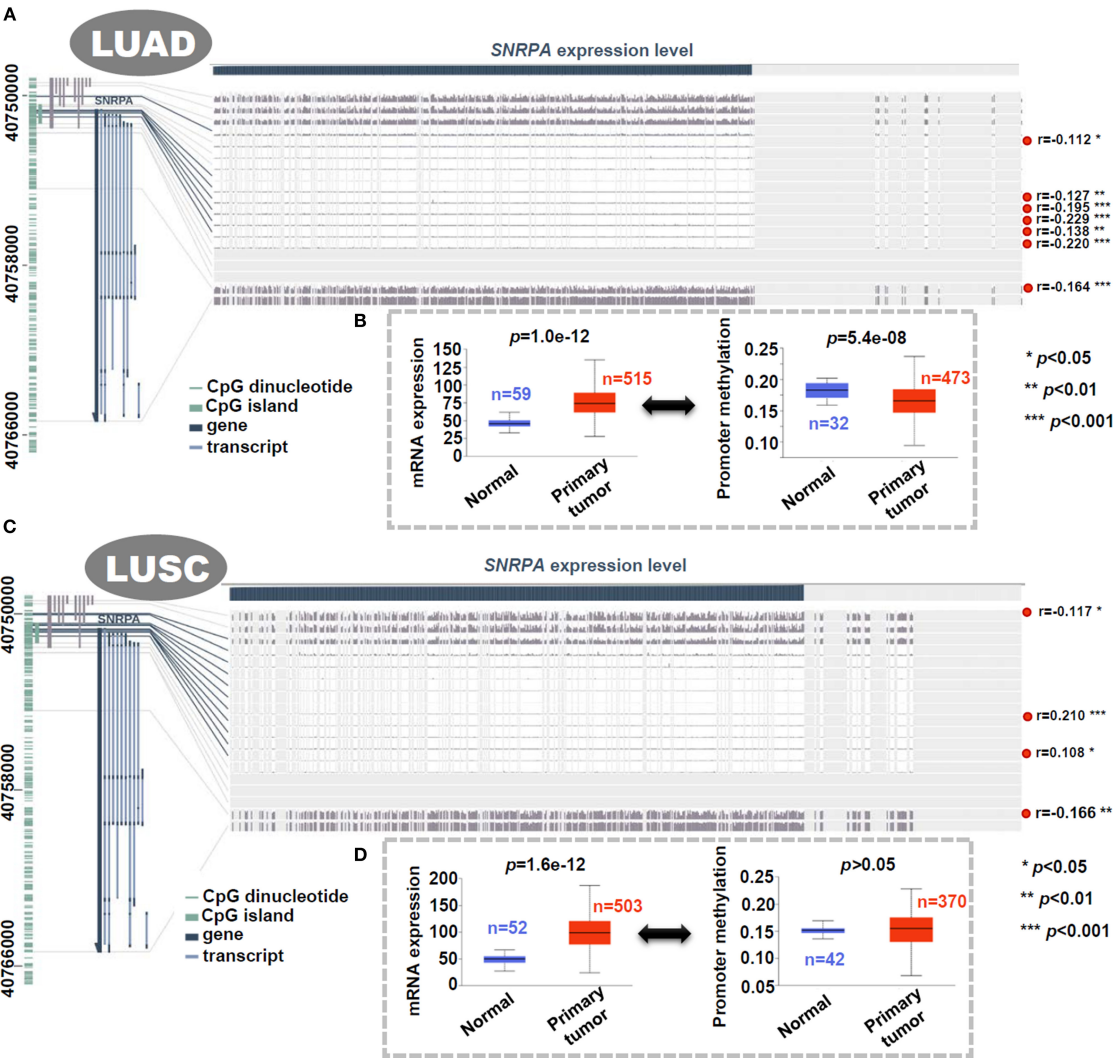
Correlation analysis between DNA methylation and expression level of *SNRPA*. We analyzed the DNA methylation status of *SNRPA* in LUAD **(A)** or LUSC **(C)** cases of TCGA through MEXPRESS. We also analyzed the difference of *SNRPA* expression or promoter methylation between the normal and primary LUAD **(B)** or LUSC **(D)** tissues through UALCAN.

Based on the methylation data of TCGA-LUAD/LUSC, the DNA methylation status of *SNRPA* was analyzed. As shown in Figure 4A, there was a negative correlation between *SNRPA* gene expression and the methylation signal values of some methylation probe sites for LUAD (*p* < 0.05, r < 0). When compared to the normal control tissues, we observed a higher mRNA expression level (Figure 4B, *p* = 1.0e−12), and a lower promoter methylation level (Figure 4B, *p* = 5.4e−08) of *SNRPA* is in the primary lung adenocarcinoma tissues. However, we did not detect similar results for LUSC (Figures 4C,D). This suggested the potential role of *SNRPA* DNA methylation in the tumorigenesis of lung adenocarcinoma.

### *SNRPA* Phosphorylation

**Figure 5:**
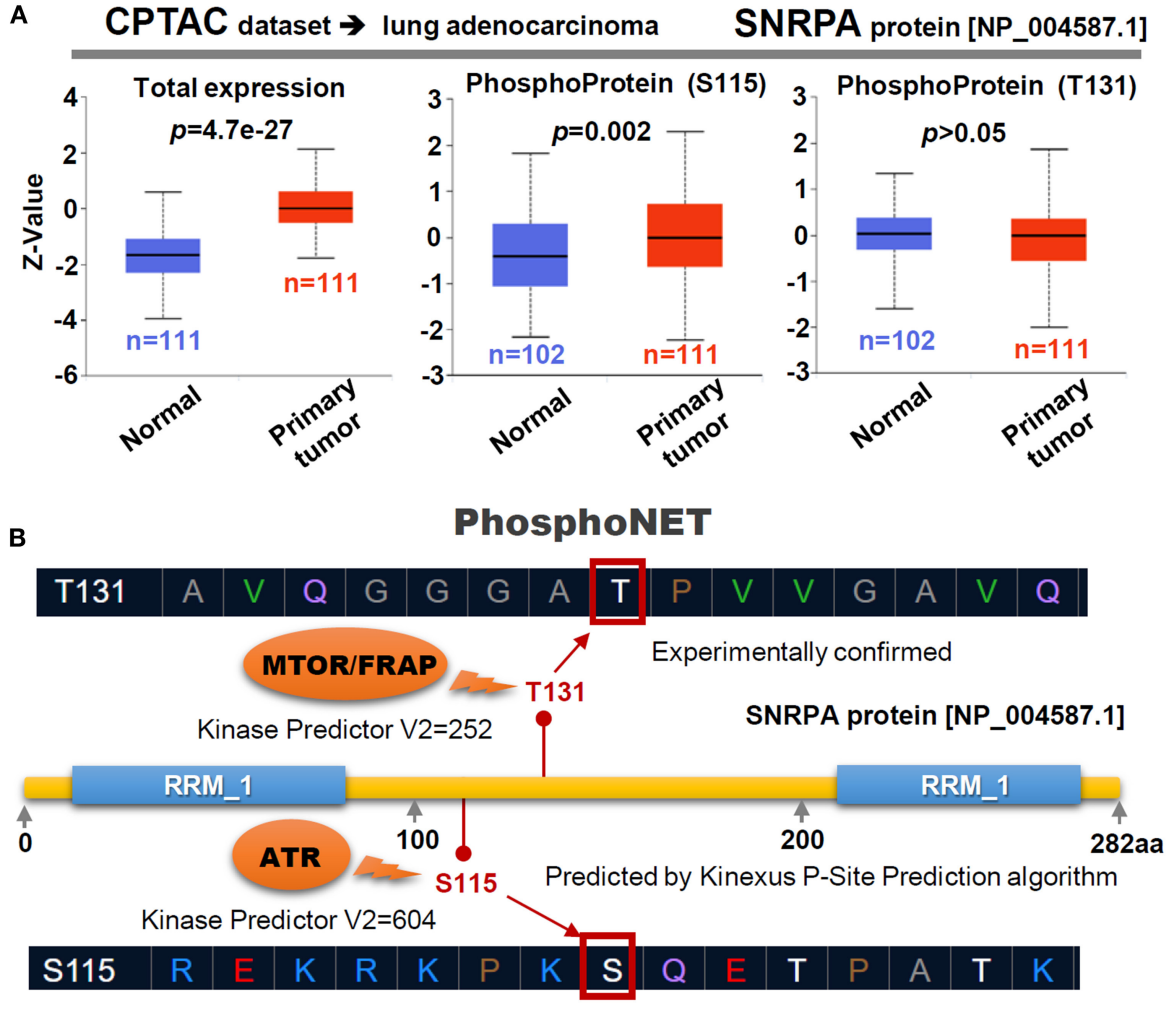
Phosphorylation of SNRPA for LUAD cases. **(A)** Targeting the LUAD dataset of CPTAC, the expression level of SNRPA total protein or phosphoprotein (NP_004587.1 S115 and T131 sites) between normal and primary tumor tissues was analyzed. **(B)** We also predicted the potential kinases of the two phosphorylation sites through a PhosphoNET tool. The score of “Kinase Predictor V2” was indicated.

Through the CPTAC database with protein expression datasets, we observed a highly expressed SNRPA protein in the primary lung adenocarcinoma tissues (Figure 5A, *p* = 4.7e−27), compared with normal tissues. Besides, the phosphorylation level at the S115 site of SNRPA protein (NP_004587.1) (Figure 5A, *p* = 0.002), but not the T131 site (*p* > 0.05), in the primary tumor tissues is higher than that in the normal tissues. We further predicted the potential kinase of two phosphorylation sites using PhosphoNET. As shown in Figure 5B, the T131 phosphorylation site of SNRPA has been experimentally confirmed (Dephoure et al., 2008), while the S115 site can be predicted by a kinexus P-site prediction algorithm. By selecting the highest “Kinase Predictor V2” score, we obtained the predicted MTOR/FRAP kinase for the T131 site and the ATR kinase for the T115 site of SNRPA protein (Figure 5B). The potential role of SNRPA phosphorylation at the S115 site in the pathological mechanism of lung adenocarcinoma merits more experimental evidence.

### *SNRPA*-Related Immune Cell Infiltration

We tried to analyze whether the *SNRPA* gene is implicated in the etiology of lung cancer through immune cell infiltration. As shown in Table 1, the *SNRPA* expression was negatively correlated with the expression of some signatures for a series of immune cells, especially the M2 macrophage in both LUAD (*r* = −0.41, *p* = 6.6e−23) and LUSC (*r* = −0.52, *p* = 4.3e−38) tissues. Furthermore, we utilized the CIBERSORT, CIBERSORT-ABS, QUANTISEQ, and XCELL algorithms of the TIMER2 approach to obtain a negative correlation between the *SNRPA* expression and the immune infiltration level of M2 macrophages (Supplementary Figure 4 all Rho < 0, *p* < 0.05). Interestingly, there was a positive correlation between the expression level of *SNRPA* and Tfh signatures (BCL6/IL21/CXCR5) in the TCGA-LUAD (Table 1, *r* = 0.23, *p* = 1.6e−10) and TCGA-LUSC (*r* = 0.13, *p* = 0.0018) cohorts. Also, we observed similar results in the immune infiltration estimation of Tfh by the algorithms of CIBERSORT and CIBERSORT-ABS (Supplementary Figure 5, Rho > 0, *p* < 0.05).

**Table 1:** Expression correlation between *SNRPA* and immune cell signatures.

**Immune cells**	**signature genes**	**LUAD**		**LUSC**	
		**r**	***p***	**r**	***p***
Monocytes	CD86/CCR2/CSF1R	−0.3	**5.1e**–**13**	−0.48	**1.1e**–**31**
M1 macrophage	NOS2/IRF5/PTGS2	−0.089	**0.038**	−0.13	**0.0019**
M2 macrophage	VSIG4/MRC1/CD163/MSR1	−0.41	**6.6e**–**23**	−0.52	**4.3e**–**38**
TAMs	CCL2/CD68/IL10	−0.35	**2.3e**–**17**	−0.49	**1.7e**–**33**
NK cell	FCGR3A/NCAM1/CD94/KIR2DL3/CD161	−0.3	**8.5e**–**13**	−0.41	**6.8e**–**23**
Neutrophils	CD66b/ITGAM/CCR7	−0.23	**1.1e**–**07**	−0.3	**8.3e**–**13**
Basophils	ENPP3/IL3RA/CCR3/PTGDR2	−0.21	**7.5e**–**07**	−0.36	**4.0e**–**18**
Eosinophils	SIGLEC8/ADGRE1	−0.3	**7.1e**–**13**	−0.37	**3.1e**–**19**
Mast cell	MITF/KIT	−0.22	**1.6e**–**07**	−0.32	**4.9e**–**14**
Dendritic cell	CD1C/ITGAX/CD83/HLA-DPB1/HLA-DRA	−0.37	**5.6e**–**19**	−0.45	**3.5e**–**28**
B cell	CD19/CR2/CD79A	−0.0012	0.98	−0.13	**0.0037**
CD8^+^ T cell	CD8A/CD8B/CD27	−0.047	0.27	−0.2	**2.4e**–**06**
Tfh	BCL6/IL21/CXCR5	0.27	**1.6e**–**10**	0.13	**0.0018**
Effector T-cell	CX3CR1/FGFBP2/FCGR3A	−0.38	**1.5e**–**19**	−0.31	**4.7e**–**13**
Exhausted T-cell	PDCD1/HAVCR2/LAG3/TIGIT/CXCL13/GZMB/CTLA4	−0.088	**0.04**	−0.12	**0.0062**

*TAMs, Tumor-associated macrophages; NK cell, Natural killer cell; Tfh, Follicular B helper T cell; LUAD, Lung adenocarcinoma; LUSC, Lung squamous cell carcinoma. Bold values mean P < 0.05*.

### Enrichment of *SNRPA*-Correlated Genes

**Figure 6:**
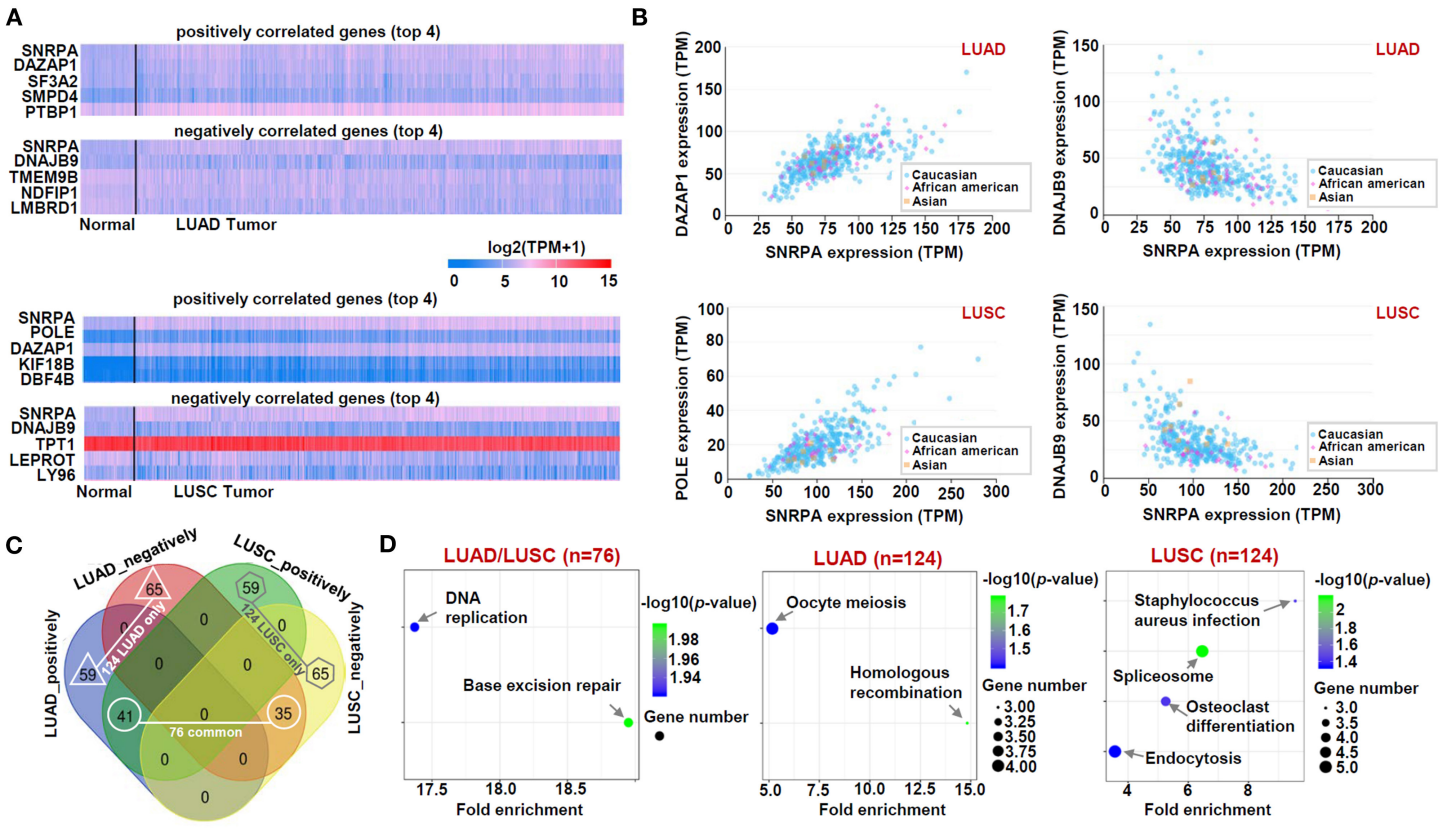
KEGG analysis of *SNRPA*-correlated genes. **(A)** The heat maps targeting the *SNRPA* positively or negatively correlated significant genes (Top 4) was shown. **(B)** The expression correlation between *SNRPA* and the selected genes (*DAZAP1, DNAJB9, POLE*) in LUAD/LUSC was analyzed, respectively. **(C)** We analyzed the difference of *SNRPA*-correlated genes between the LUAD and LUSC through a Venn tool. **(D)** Based on three gene lists, namely “LUAD/LUSC common” (*n* = 76), “LUAD only” (*n* = 124), and “LUSC only” (*n* = 124) genes, KEGG pathway analyses were performed.

Finally, based on the datasets of the TCGA-LUAD/LUSC cohorts, we screened out a group of *SNRPA* expression-correlated positively (e.g., *DAZAP1, SF3A2, SMPD4*, and *PTBP1*, etc.) or negatively (e.g., *DNAJB9, TMEM9B, NOFIP1*, and *LMBRD1*, etc.) genes in Figure 6A, using UALCAN approach. Our correlation analyses with the marker of the race indicated a strong expression correlation between *SNRPA* and the selected *DAZAP1, DNAJB9*, or *POLE* gene (Figures 6A,B). Then, we conducted an intersection analysis to obtain three gene lists, namely “LUAD/LUSC common” (*n* = 76), “LUAD only” (*n* = 124), and “LUSC only” (*n* = 124) genes (Figure 6C), for a series of *SNRPA*-related enrichment analysis.

**Figure 7:**
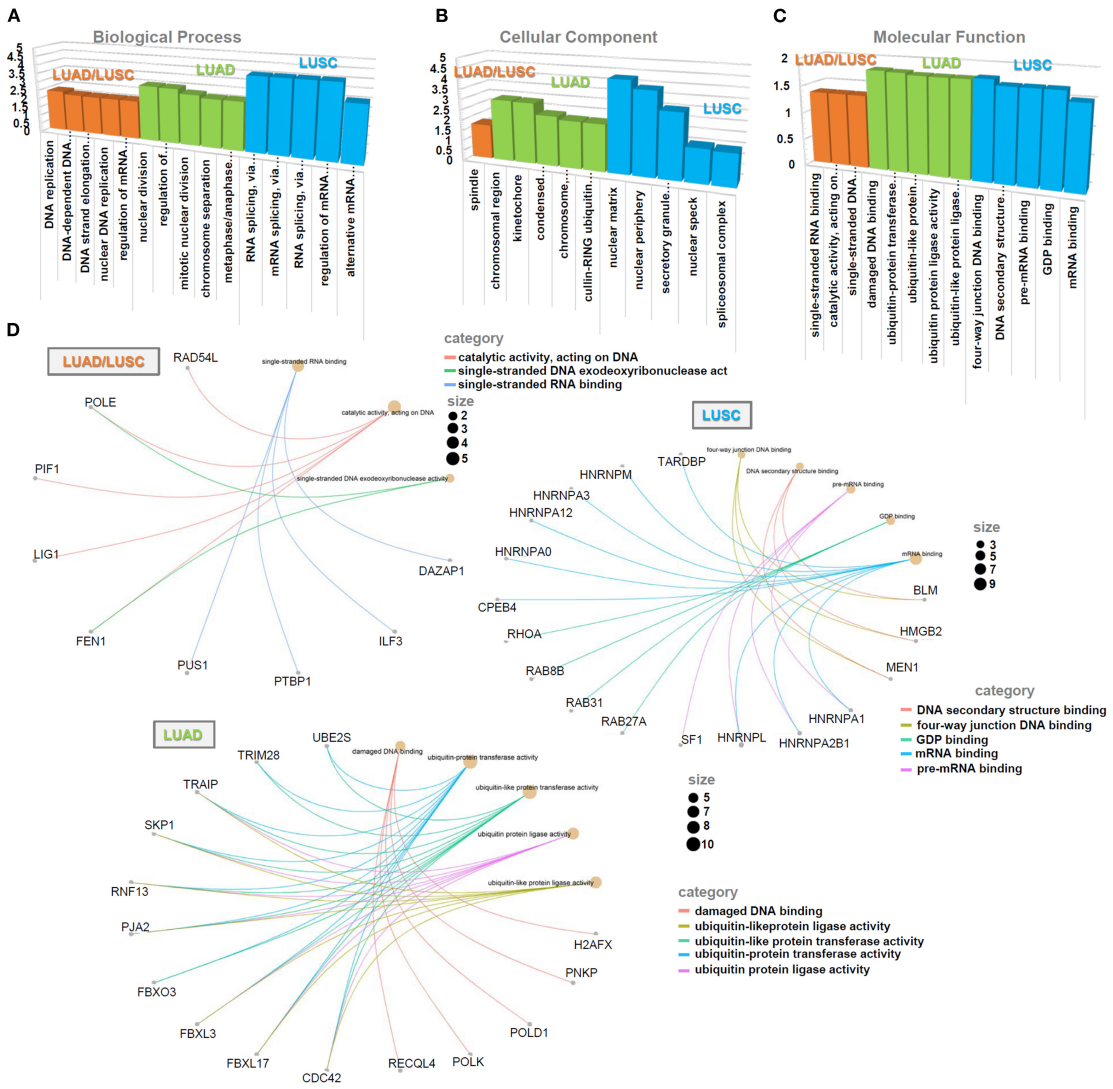
GO analysis of *SNRPA*-correlated genes. Based on three gene lists (“LUAD/LUSC common,” “LUAD only,” and “LUSC only”), the enriched information of biological process **(A)**, cellular component **(B)**, and molecular function **(C)** was provided. **(D)** The cnetplots of molecular function during the GO analysis of different groups were provided, respectively.

The data of the KEGG pathway (Figure 6D), GO (Figures 7A–C) and GSEA (Supplementary Figures 6, 7) analyses showed the distinct enriched pathways among the different groups. The DNA/RNA metabolism-related cellular pathways or biological processes (e.g., DNA replication, base excision repair, regulation of mRNA metabolic process, single-stranded RNA binding, etc.) were detected in the LUAD/LUSC common group (Figures 6D, 7A–C; Supplementary Figures 6A,B, 7A,B). Cell cycle and ubiquitin mechanism-related issues (e.g., oocyte meiosis, nuclear division, G2M checkpoint, cullin-RING ubiquitin ligase complex, ubiquitin-protein transferase activity, ubiquitin-protein ligase activity, etc.) were observed for the LUAD group; however, the RNA splicing-related cellular issues (e.g., spliceosome, RNA splicing, pre-mRNA binding, alternative mRNA splicing, etc.) were detected for the LUSC group (Figures 6D, 7A–C; Supplementary Figures 6C,D, 7C,D). Figure 7D presents the cnetplots of molecular function during the GO analysis of different groups. These suggested there may exist distinct molecular mechanisms regarding the role of *SNRPA* in the pathogenesis of LUAD and LUSC.

## Discussion

Through analyzing the datasets within TCGA and GEO databases, our study aimed to investigate the potential role of SNRPA expression, modification, or genetic mutation in the prognosis of lung adenocarcinoma (LUAD) and lung squamous cell carcinoma (LUSC) of NSCLC. But the type of SCLC was not included. LUAD and LUSC exhibit different pathological characteristics, clinical characteristics, and treatment strategies (Duffy and O'Byrne, 2018; Testa et al., 2018; Friedlaender et al., 2019). Our findings showed that the SNRPA may be related to the pathogenesis of LUAD and LUSC through distinct molecular mechanisms.

Using a series of integrated analyses, we first identified a high expression level of *SNRPA* in both LUAD and LUSC tissues, compared with the normal controls. Even though there was no statistical correlation between the *SNRPA* gene and the pathological stage of LUAD, we observed that the *SNRPA* exhibits an upward trend with the pathological stage I~IV, and the *SNRPA* expression was also correlated with the “pathologic n” factor. In addition, we found that the LUAD cases with the stages (II, III/IV) or high *SNRPA* expression were correlated with a poor overall clinical survival prognosis, through our multivariate COX regression analysis. The high *SNRPA* expression is also associated with the poor first- and post- progression survival prognosis of LUAD cases. Based on the data of the TCGA-LUSC cohort, although pathological stage factors were statistically correlated with the *SNRPA* expression and the OS prognosis of cases, there is a very limited number of LUSC cases with stage IV, which even showed a relatively low expression level of SNPRA. We did not detect a correlation between *SNRPA* gene expression and clinical prognosis of cases in TCGA, but there existed the worse clinical OS outcomes for the LUSC cases with highly expressed *SNRPA* in GEO datasets. Surprisingly, the high *SNRPA* expression was detected to be linked to a better first-progression prognosis of lung cancer cases in GEO. How to explain this point? It is likely that the LUSC cases with a lowly expressed *SNRPA* are prone to suffer from the first disease progression. Once the progression occurred, there is no difference in survival time between the high-expression and low-expression groups of *SNRPA*. However, we sense that there were only 37 cases in the “Low-expression” group, and the clinical factors were not adjusted, due to the very limited data. In addition, considering the lack of strong evidence regarding the association between the *SNRPA* expression and clinical prognosis of LUSC cases in TCGA, the expression data of more LUSC patients with strict quality control and complete clinical information collection are required to confirm the prognostic value of SNRPA in LUSC.

It is known that tobacco smoking is linked to the occurrence of lung cancer (Klebe et al., 2019). In our subgroup analysis, we found that high *SNRPA* expression was correlated with a poor clinical prognosis of both smokers and non-smokers. Thus, the “smoking” factor may not be essential for the involvement of *SNRPA* in the pathogenesis of lung cancer. Our subgroup analyses of small cell lung cancer by some clinical factors showed no difference in the correlations between high *SNRPA* expression and poor clinical prognosis of OS, FP, and PPS between the smokers and non-smokers with lung cancer. The factor of smoking history may not be involved in the mechanism of *SNRPA* in the pathogenesis of lung cancer. Besides, we note that the associations between high *SNRPA* expression and poor OS, FP, and PPS prognosis of lung cancer patients were mainly detected in the pathological stage I group, suggesting the potential role of *SNRPA* in the early stages of lung cancer. Interestingly, there was correlation between low *SNRPA* expression and poor post-progression survival prognosis of lung cases treated with chemotherapy drugs (HR = 0.57, *p* = 0.035). More subjects are needed for a meaningful analysis of the potential relationship between *SNRPA* expression and chemotherapy drug sensitivity.

The occurrence and pathogenesis of lung cancer are closely linked to the issues of genetic mutation (Castellanos et al., 2017; Hou et al., 2017; Testa et al., 2018; Friedlaender et al., 2019), DNA methylation (Shi et al., 2019; Soca-Chafre et al., 2019), and immune cell infiltration (Bremnes et al., 2011; Zheng et al., 2017; Zhang et al., 2019). Herein, we found that the genetic alteration of *SNRPA* was associated with the overall survival prognosis of LUSC cases, but not LUAD cases. However, it should be noted that the genetic alteration frequency of both LUAD and LUSC is not higher than 5%, which greatly reduces the likelihood of the involvement of *SNRPA* genetic mutations in the pathogenesis of LUAD. In addition, we detected a negative correlation between *SNRPA* gene expression and its promoter methylation for the LUAD cases. However, this phenomenon is not obvious for LUSC cases. Also, given a low methylation level of *SNRPA*, the DNA methylation factor may slightly contribute to the complicated mechanisms of *SNRPA* in lung cancer. With regards to immune cell infiltration, *SNRPA* expression was negatively correlated with the infiltration level of M2 macrophage but positively correlated with the infiltration level of follicular B helper T cell, in both LUAD and LUSC. This provides a novel perspective for investigating the mechanism of *SNRPA* in the pathogenesis of NSCLC.

In this study, we utilized two different analyzing strategies for the enrichment of the *SNRPA*-correlated genes. On the one hand, after obtaining a series of *SNRPA*-related genes by UALCAN approach and intersection analysis, respectively, GO and KEGG enrichment analyses were conducted by DAVID, “clusterProfiler” and “ggplot2” R package, etc. On the other hand, we performed a GESA analysis, using the expression matrices of TCGA-LUAD/LUSC cohorts grouped by the median value of *SNRPA* expression. We obtained similar conclusions. The DNA damage repair of cells under various stress conditions is closely related to the occurrence of tumors (Basu, 2018; Sen et al., 2018). DNA/RNA metabolism-related cellular pathways or biological processes, especially the base excision repair, were both involved in the potential role of *SNRPA* in the pathogenesis of LUAD and LUSC. Considering the essential role of SNRPA in the cellular pre-mRNA splicing process (Singh and Singh, 2019) and the association between pre-mRNA processing factors and DNA damage response (Montecucco and Biamonti, 2013), it is meaningful to analyze the potential functional link of SNPRA with DNA damage repair pathway during tumorigenesis. Further, cell cycle and ubiquitin mechanism-associated issues may be involved in the LUAD pathogenesis, whereas RNA splicing-related cellular issues seem to be important for LUSC. More experiment results are needed for the confirmation of distinctive *SNRP*A effects on the different types of lung cancer.

The S115 phosphorylation site of SNRPA could be predicted by a kinexus P-site prediction algorithm. After analyzing the LUAD data of CPTAC, we observed a higher phosphorylation level of SNRPA at the S115 site in the primary tumor than the controls. ATR is the potential kinase of SNRPA. Even though T131 phosphorylation sites of SNRPA protein have been experimentally confirmed (Dephoure et al., 2008), we did not detect the statistical difference in the phosphorylation level at this site between normal and tumor tissues. Due to the lack of the LUSC dataset within CPTAC, we did not analyze the phosphorylation status of SNRPA for LUSC. Additionally, we predicted that there are many other phosphorylation sites (e.g., Y31, T118, etc.), ubiquitylation sites (e.g., K20, K50, K88, etc.), acetylation sites (e.g., K122, K80, K96, etc.) of SNRPA protein (data not shown). More sample sizes, protein expression, clinical and basic experimental data are needed for an in-depth investigation.

## Conclusion

Based on the available online datasets, we first identified the potential prognostic value of SNRPA in non-small cell lung cancer. SNRPA may function distinctively in the pathogenesis of lung adenocarcinoma and lung squamous cell carcinoma tissue. More in-depth cell molecular experiments are required.

## Data Availability Statement

The original contributions presented in the study are included in the article/Supplementary Material, further inquiries can be directed to the corresponding author.

## Author Contributions

MY and CY designed the research and wrote the manuscript. MY and XC performed the expression, survival curve, and genetic alteration analyses. CY and YW performed the DNA methylation, phosphorylation, and immune cell infiltration analyses. CY, XC, and YW performed the *SNRPA*-correlated gene analysis. All authors have read and approved the manuscript.

## Conflict of Interest

The authors declare that the research was conducted in the absence of any commercial or financial relationships that could be construed as a potential conflict of interest.

## Supplementary Material

Supplementary Figure 1 *SNRPA* expression of LUAD/LUSC and corresponding para-carcinoma tissues. We analyzed the expression difference of *SNRPA* between lung cancer and corresponding para-carcinoma tissues. **(A)** *n* = 57 for LUAD pair; **(B)** *n* = 49 for LUSC pair. The *p*-value of a Wilcoxon test was shown.

Supplementary Figure 2 Survival curve analysis of *SNRPA* (TCGA). We performed the overall survival (OS) analyses of TCGA-LUAD **(A)** and TCGA-LUSC **(B)**, according to the expression level of the *SNRPA* gene, through GEPIA2.

Supplementary Figure 3 Survival curve analysis of *SNRPA* (GEO). We utilized a Kaplan-Meier plotter to perform the overall survival (OS) **(A)**, first-progression (FP) **(B)**, post-progression survival (PPS) **(C)** analyses of SNRPA for LUAD and LUSC.

Supplementary Figure 4 Correlation analysis between *SNRPA* expression and immune infiltration of M2 macrophage. The algorithms of CIBERSORT, CIBERSORT-ABS, QUANTISEQ, and XCELL were used for LUAD **(A–D)** and LUSC **(E–H)**, respectively.

Supplementary Figure 5 Correlation analysis between *SNRPA* expression and immune infiltration of Tfh. Two algorithms (CIBERSORT and CIBERSORT-ABS) were used for LUAD **(A,B)** and LUSC **(C,D)**, respectively.

Supplementary Figure 6 GSEA analysis of *SNRPA* for LUAD. **(A)** DNA replication; **(B)** Base excision repair; **(C)** Cell cycle; **(D)** G2M checkpoint.

Supplementary Figure 7 GSEA analysis of *SNRPA* for LUSC. **(A)** DNA replication; **(B)** Base excision repair; **(C)** Spliceosome; **(D)** Spliceosomal complex.

Supplementary Table 1 Subgroup analysis regarding the correlation of SNRPA expression and prognosis of lung cancer cases.
