# Supplementary figures and images for: Investigation on Potential Correlation Between Small Nuclear Ribonucleoprotein Polypeptide A and Lung Cancer
Source: Front Genet. 2021 Jan 21;11:610704. doi: 10.3389/fgene.2020.610704 (PMC7859448; doi:10.3389/fgene.2020.610704)

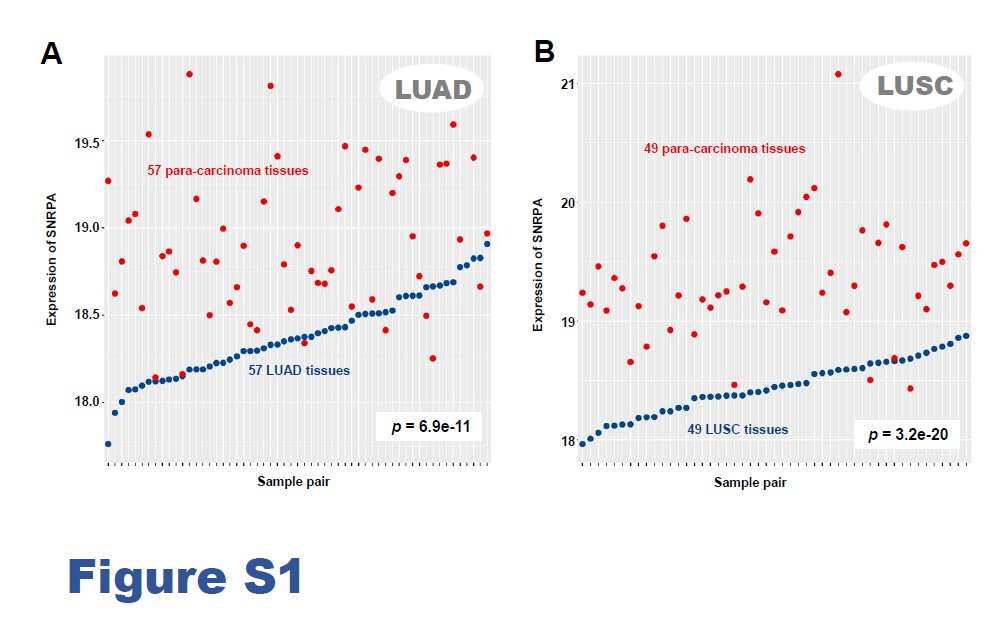

Supplement: Supplementary Figure 1 — SNRPA expression of LUAD/LUSC and corresponding para-carcinoma tissues. We analyzed the expression difference of SNRPA between lung cancer and corresponding para-carcinoma tissues. (A) n = 57 for LUAD pair; (B) n = 49 for LUSC pair. The p-value of a Wilcoxon test was shown. [file Image_1.TIF]

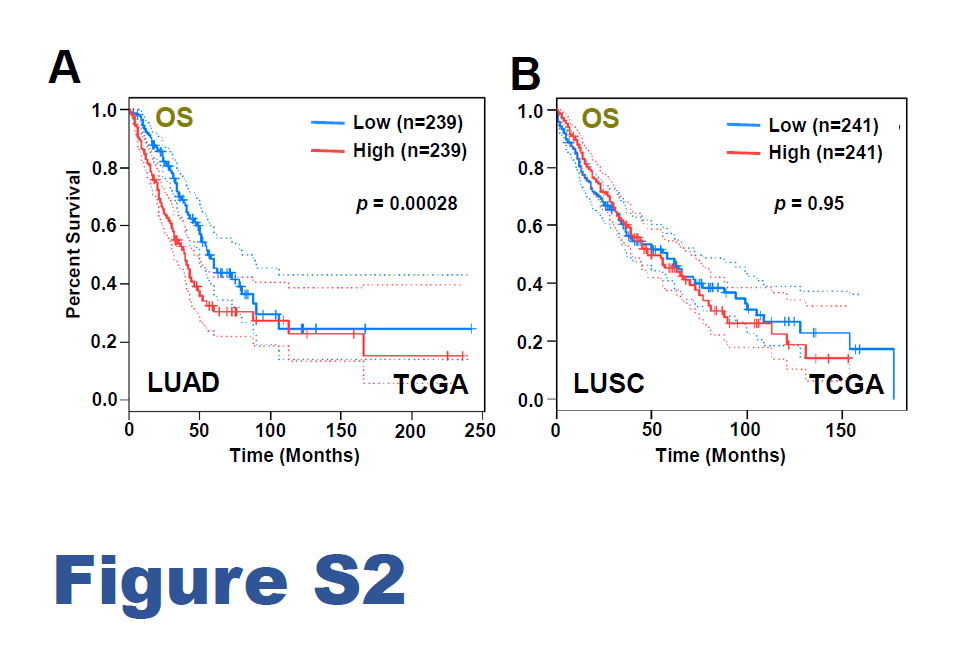

Supplement: Supplementary Figure 2 — Survival curve analysis of SNRPA (TCGA). We performed the overall survival (OS) analyses of TCGA-LUAD (A) and TCGA-LUSC (B), according to the expression level of the SNRPA gene, through GEPIA2. [file Image_2.TIF]

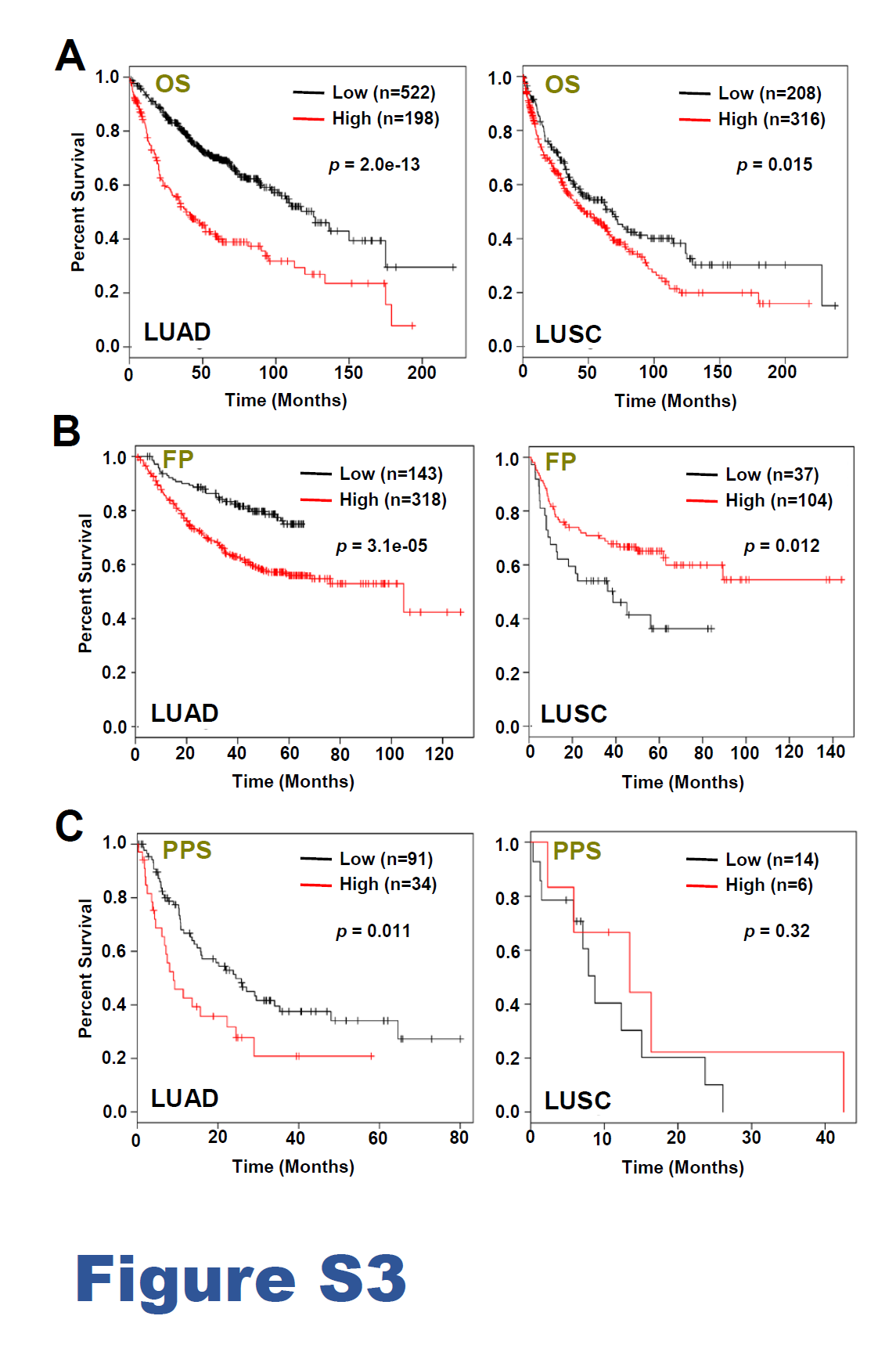

Supplement: Supplementary Figure 3 — Survival curve analysis of SNRPA (GEO). We utilized a Kaplan-Meier plotter to perform the overall survival (OS) (A), first-progression (FP) (B), post-progression survival (PPS) (C) analyses of SNRPA for LUAD and LUSC. [file Image_3.TIF]

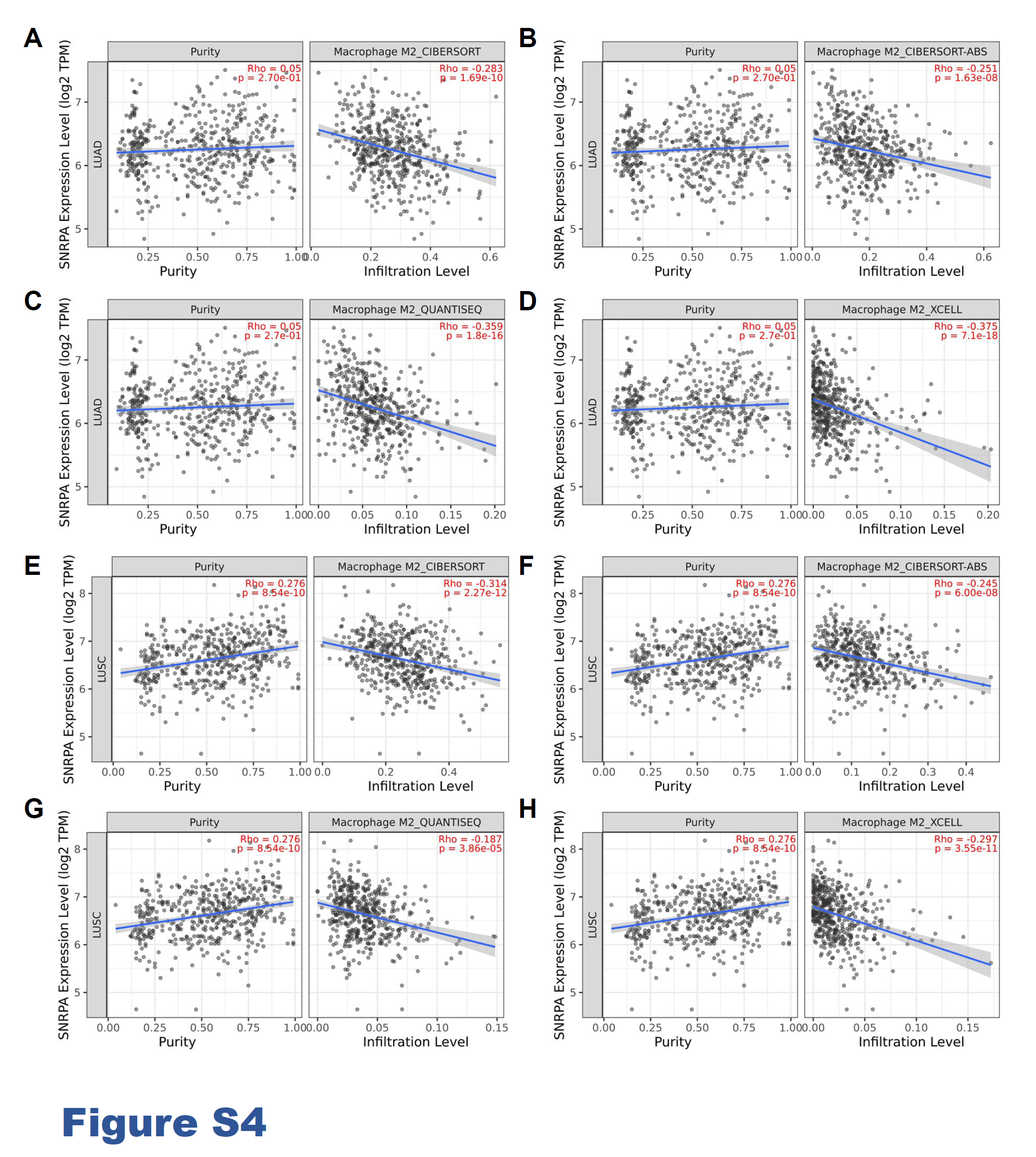

Supplement: Supplementary Figure 4 — Correlation analysis between SNRPA expression and immune infiltration of M2 macrophage. The algorithms of CIBERSORT, CIBERSORT-ABS, QUANTISEQ, and XCELL were used for LUAD (A–D) and LUSC (E–H), respectively. [file Image_4.TIF]

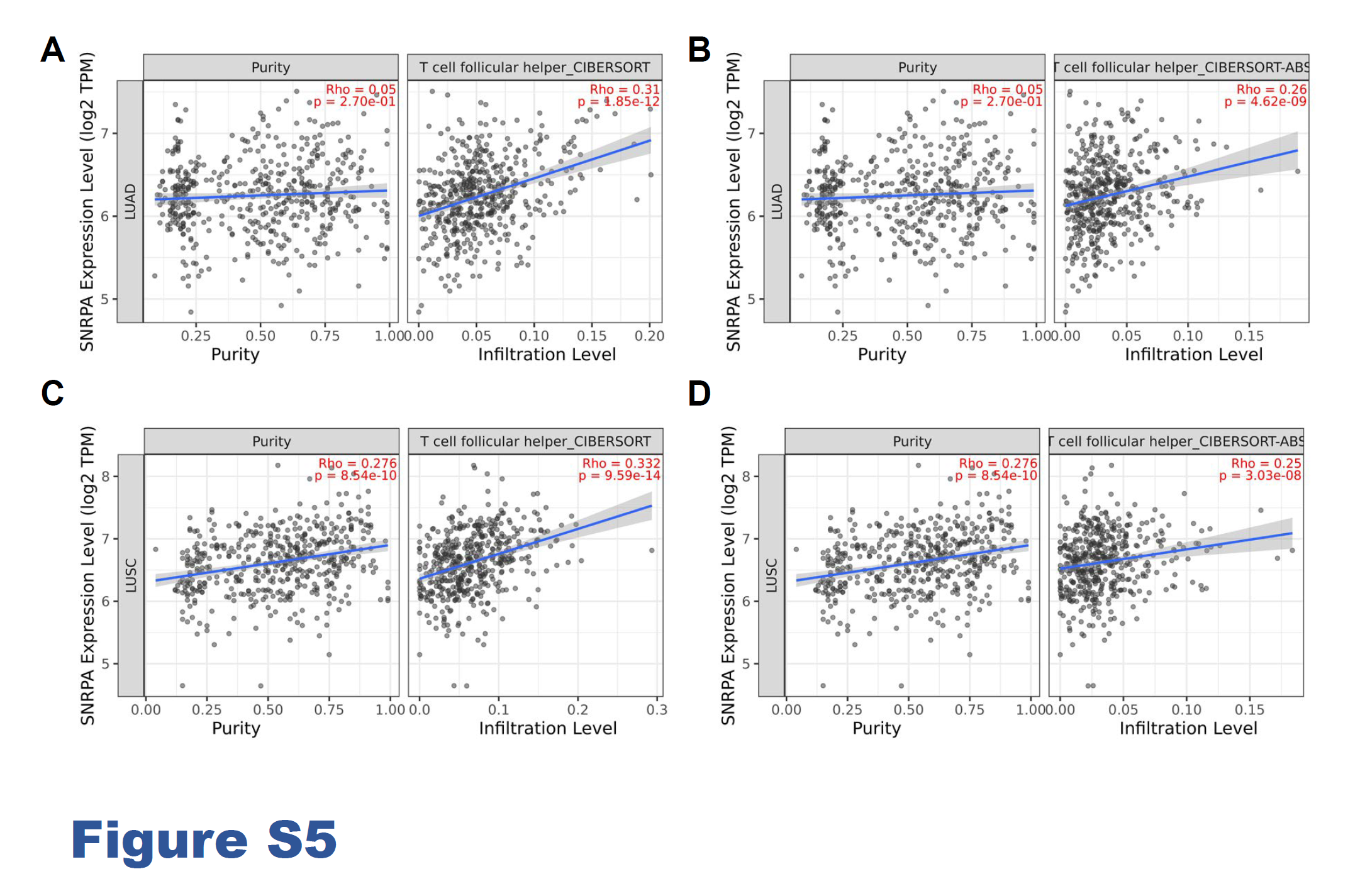

Supplement: Supplementary Figure 5 — Correlation analysis between SNRPA expression and immune infiltration of Tfh. Two algorithms (CIBERSORT and CIBERSORT-ABS) were used for LUAD (A,B) and LUSC (C,D), respectively. [file Image_5.TIF]

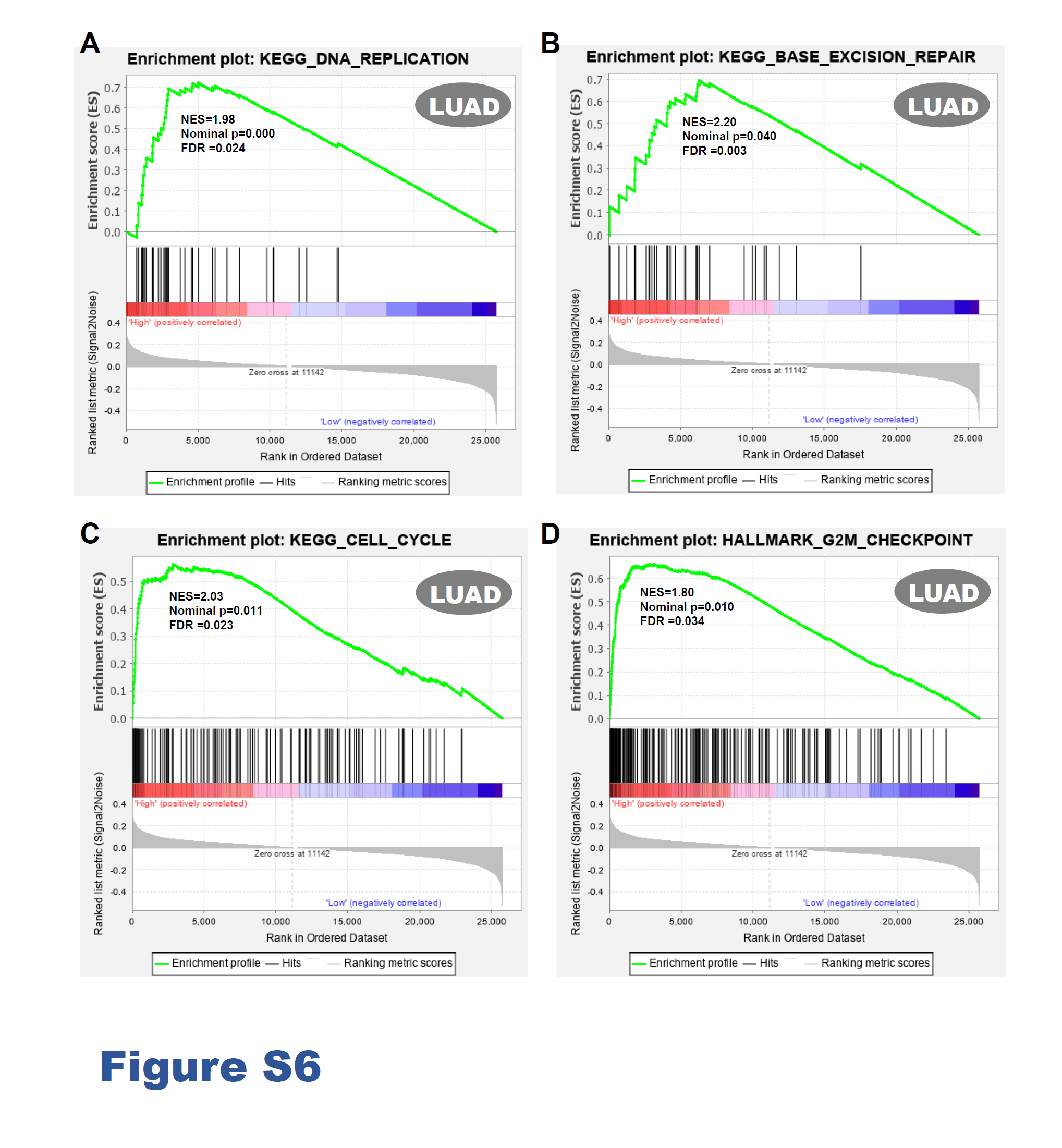

Supplement: Supplementary Figure 6 — GSEA analysis of SNRPA for LUAD. (A) DNA replication; (B) Base excision repair; (C) Cell cycle; (D) G2M checkpoint. [file Image_6.TIF]

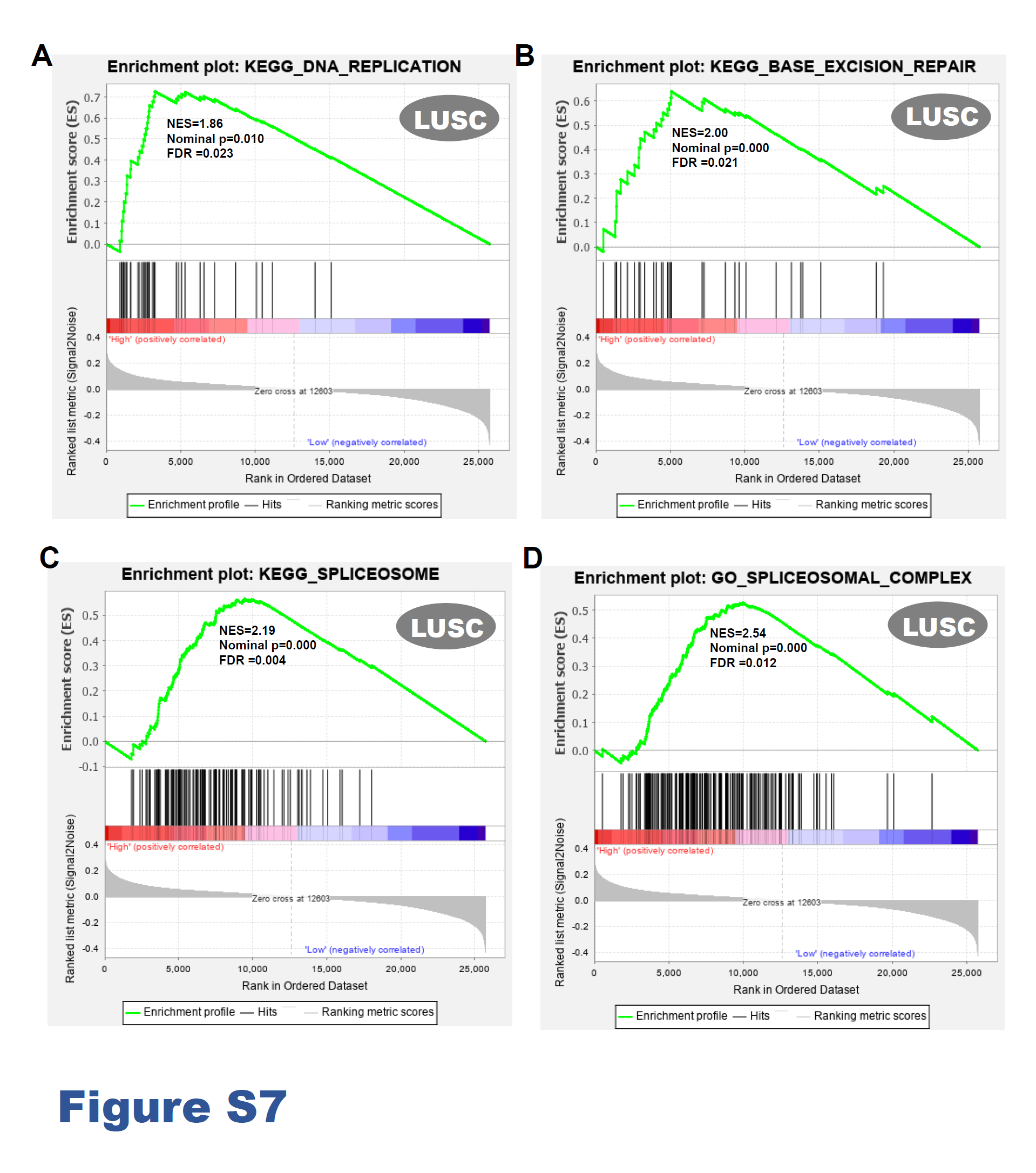

Supplement: Supplementary Figure 7 — GSEA analysis of SNRPA for LUSC. (A) DNA replication; (B) Base excision repair; (C) Spliceosome; (D) Spliceosomal complex. [file Image_7.TIF]
