# Supplementary material for: Investigation on Potential Correlation Between Small Nuclear Ribonucleoprotein Polypeptide A and Lung Cancer
Source: Front Genet. 2021 Jan 21;11:610704. doi: 10.3389/fgene.2020.610704 (PMC7859448; doi:10.3389/fgene.2020.610704)
Supplement: Supplementary Table 1 — Subgroup analysis regarding the correlation of SNRPA expression and prognosis of lung cancer cases. [file Table_1.DOCX]

## TABLE S1 | Subgroup analysis regarding the correlation of *SNRPA* expression and prognosis of lung cancer cases.

| **Subgroup** | **Group** | **Sample size** | **OS** | | **FP** | | **PPS** | |
| --- | --- | --- | --- | --- | --- | --- | --- | --- |
|  |  |  | **HR** | ***p*** | **HR** | ***p*** | **HR** | ***p*** |
| **gender** | female | 818 | 2.05 | **6.0e-07** | 1.78 | **0.00049** | 1.61 | **0.02** |
|  | male | 1387 | 1.72 | **2.1e-11** | 1.82 | **0.00042** | 1.41 | 0.065 |
| **smoking history** | exclude those never smoked | 970 | 1.55 | **4.4e-05** | 1.45 | **0.0029** | 1.37 | **0.039** |
|  | only those never smoked | 247 | 3.22 | **2.4e-05** | 2.85 | **0.00059** | 2.19 | **0.019** |
| **stage** | stage I | 652 | 3.18 | **1.1e-16** | 1.99 | **0.0073** | 3.48 | **6.1e-05** |
|  | stage II | 320 | 2.39 | **2.0e-06** | 0.78 | 0.35 | 1.89 | 0.076 |
|  | stage III | 70 | 1.56 | 0.2 | - | **-** | - | **-** |
| **grade** | grade I | 202 | 1.24 | 0.24 | 1.47 | 0.11 | 1.33 | 0.26 |
|  | grade II | 310 | 0.81 | 0.22 | 0.78 | 0.24 | 0.78 | 0.33 |
|  | grade III | 77 | 1.97 | 0.074 | 1.83 | 0.26 | 2.85 | **0.046** |
| **AJCC stage t** | t1 | 475 | 1.62 | **0.0012** | 0.67 | 0.2 | 2.54 | **0.0059** |
|  | t2 | 686 | 1.22 | 0.078 | 1.32 | 0.074 | 0.75 | 0.12 |
|  | t3 | 99 | 1.37 | 0.29 | 2.91 | **0.043** | - | **-** |
|  | t4 | 48 | 2.08 | **0.023** | - | - | - | -- |
| **AJCC stage n** | n0 | 863 | 1.28 | **0.035** | 1.27 | 0.19 | 1.34 | 0.17 |
|  | n1 | 296 | 1.61 | **0.031** | 1.54 | 0.066 | 1.98 | **0.014** |
|  | n2 | 113 | 1.34 | 0.15 | 1.36 | 0.37 | 0.44 | **0.048** |
| **AJCC stage m** | m0 | 818 | 1.94 | **1.4e-09** | 1.96 | **0.012** | 1.86 | 0.065 |
| **surgery** | only surgical margins negative | 730 | 3.72 | **6.3e-13** | 3.23 | **3.7e-12** | 1.58 | **0.0044** |
| **radiotherapy** | no | 276 | 0.67 | **0.035** | 0.74 | 0.13 | 1.88 | **0.0055** |
|  | yes | 73 | 0.62 | 0.083 | 1.61 | 0.08 | 0.61 | 0.13 |
| **chemotherapy** | no | 317 | 1.29 | 0.16 | 0.56 | **0.0033** | 1.71 | **0.03** |
|  | yes | 178 | 2.03 | **0.00051** | 2.62 | **4.9e-06** | 0.57 | **0.035** |

HR, hazard ratio; AJCC，American Joint Committee on Cancer; OS, overall survival; FP, first-progression; PPS, post-progression survival; -, no data.
